# Intussusception of the small bowel secondary to malignant metastases in two 80-year-old people: a case series

**DOI:** 10.1186/1752-1947-5-176

**Published:** 2011-05-11

**Authors:** Charalambos Spiridis, Apostolos Kambaroudis, Achilleas Ntinas, Savvas Papadopoulos, Athanasios Papanicolaou, Thomas Gerasimidis

**Affiliations:** 1 5th Surgical Clinic, Hippokrateion General Hospital, 49 Konstantinoupoleos str, PO 54642, Thessaloniki, Greece; 2 16 Sokratous str, PO 56123, Thessaloniki, Greece; 3 Department of Pathologic Anatomy, Hippokrateion General Hospital, 49 Konstantinoupoleos str, PO 54642, Thessaloniki, Greece

## Abstract

**Introduction:**

Small bowel intussusception is rare in adults and accounts for one percent of all bowel obstructions. Malignancy is the etiologic agent in approximately 50 percent of all cases.

**Case presentation:**

Our first patient was an 80-year-old Caucasian woman with signs and symptoms of intermittent bowel obstruction for the last 12 months. Pre-operative investigation by abdominal computed tomography scanning revealed an obstruction at the ileocecal valve. Exploratory laparotomy revealed an ileocecal intussusception. She underwent an enterectomy. Histological examination showed metastatic breast cancer (lobular carcinoma). Our patient had previously undergone a mastectomy due to carcinoma three years earlier.

Our second patient was an 80-year-old Caucasian man with signs and symptoms of acute bowel obstruction. Pre-operative investigation by abdominal computed tomography scanning showed an intussusception in the proximal part of the small bowel. Exploratory laparotomy revealed a jejunojejunal intussusception. He underwent an enterectomy. Histological examination showed metastatic melanoma. Our patient had a prior history of a primary cutaneous melanoma which was excised two years ago.

**Conclusion:**

Pre-operative determination of the etiologic agent of intussusception in the small bowel in adults is difficult. Although a computed tomography scan is very helpful, the diagnosis of intussusception is made by exploratory laparotomy and histological examination defines the etiologic agent. A prior malignancy in the patient's history must be taken under consideration as a possible cause of intussusception.

## Introduction

Intussusception is the most common (1.5-four cases per 1000 live births) [1] cause of small bowel obstruction and possible enteric ischemia in children but it is rare in adults. There are significant differences in regard to location, etiology, presentation and management of intussusception between adults and children. In adults, the small bowel is the most common location of intussusception and in 90% of cases the lead point is a benign or malignant tumor [2]. Clinical presentation is variable and can be acute, intermittent or chronic, a fact that increases the difficulty of preoperative diagnosis [2].

The aim of this paper is to determine the difficulties and problems of a precise pre-operative diagnosis and the management of intussusception in adults. We describe two cases of intussusception secondary to malignant metastases.

## Case presentation

### First case

An 80-year-old Caucasian woman was admitted to our department with acute abdomen. She presented with abdominal pain, no passage of flatus or stool, and vomiting. In the last year she had three episodes of intermittent bowel obstruction and a weight loss of 22 kilograms, for which she was treated conservatively. Our patient had undergone a left mastectomy for lobular carcinoma of the breast three years ago. She had no history of previous abdominal operations. During the last year she presented with bone metastases (diagnosed by bone scintigraphy, which was negative for abdominal disease) and she was under continuous administration of letrozole and zoledronic acid.

**Figure 1:**
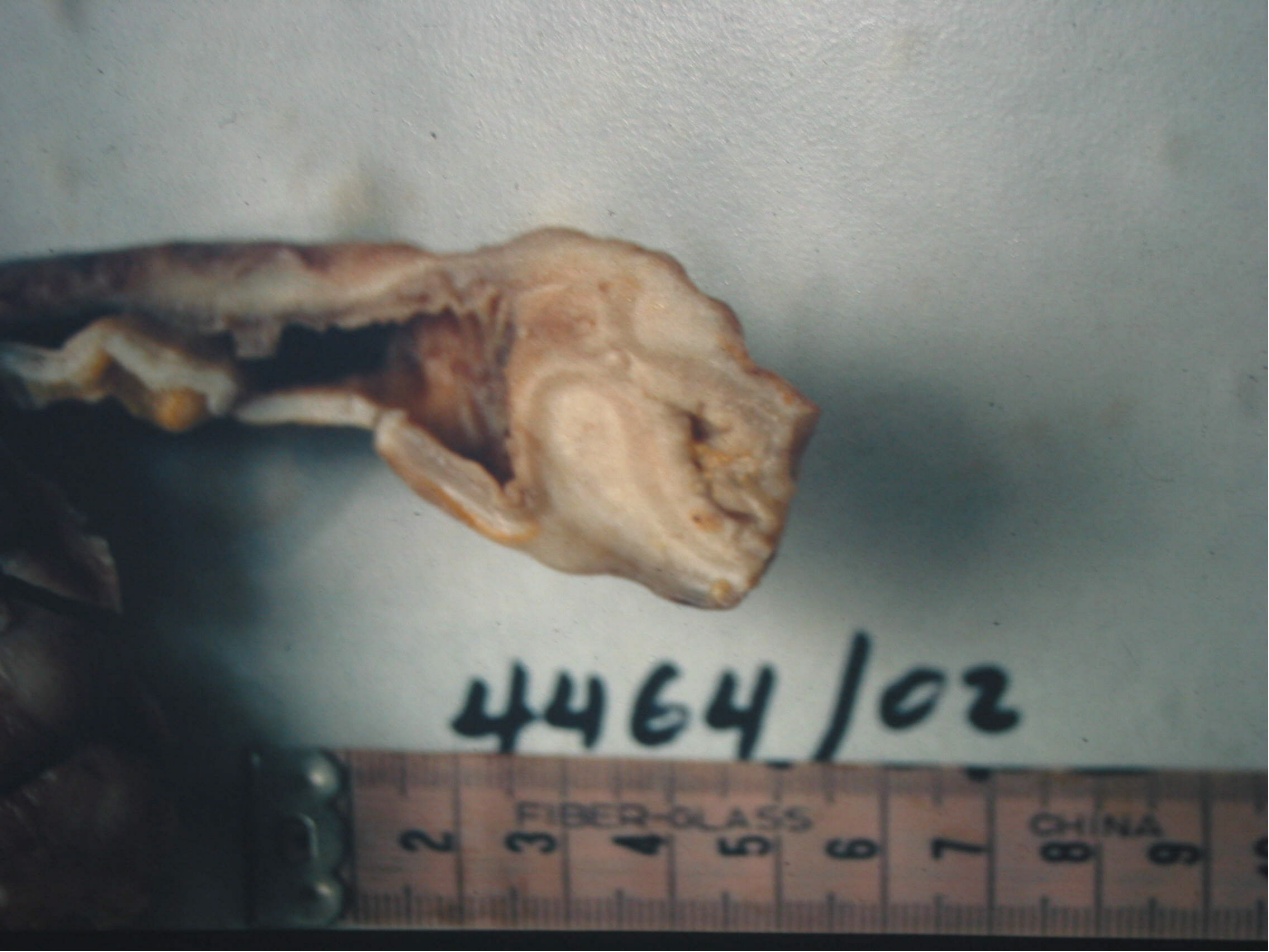
**The surgical preparation in oblong cross-section**.

**Figure 2:**
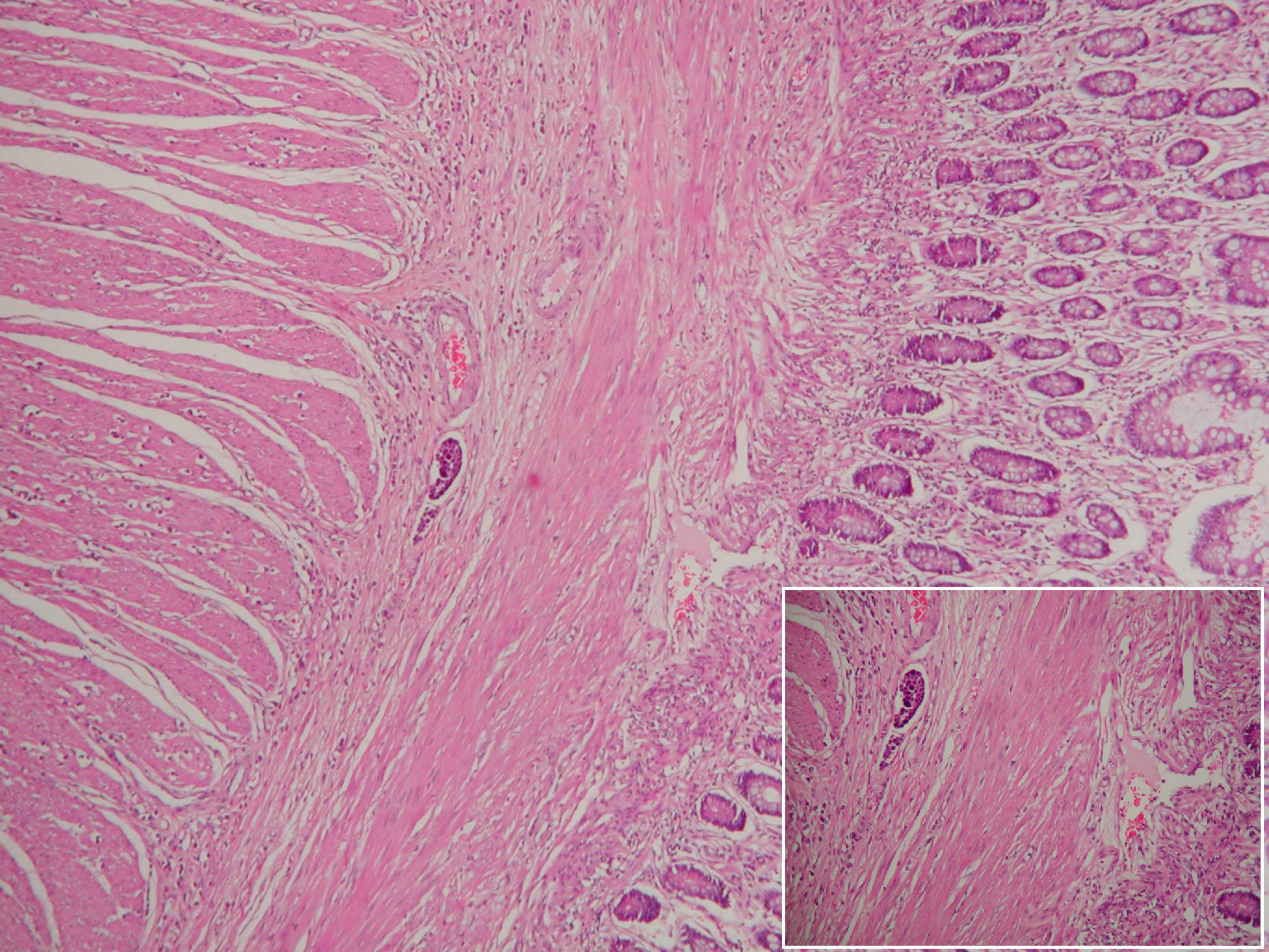
**Microscopic slide of the surgical preparation, H-E stain, ×40**. Diffuse infiltration of the muscularis layer of the intestine from lobular carcinoma of the breast with obvious neoplastic embolus in a lymphatic vessel. *Inset: *The neoplastic embolus and the infiltration of the intestinal wall in higher magnification (H-E ×100).

On admission, her abdominal X-rays showed intestinal air-fluid levels and an abdominal computed tomography (CT) scan showed distended intestinal loops and thickening of her intestinal wall. It showed no abdominal masses or other evidence of peritoneal carcinomatosis, but was suggestive of an obstruction at the ileocecal valve. An ileal intussusception was found during laparotomy. Her small bowel was dilated from the ligament of Treitz to approximately 10 cm proximal to the ileocecal valve. The cause of intussusception was an intraluminal mass 3 × 4 cm in size (Figure 1). No hepatic masses were found but some nodules were palpable in the mesentery. Approximately 30 cm of ileum were resected and continuity was re-established with an end-to-end anastomosis. A histological examination demonstrated multiple foci of lobular carcinoma of the breast (Figure 2). Our patient's recovery was uneventful and she is under meticulous follow up and drug administration (letrozole and zoledronic acid).

### Second case

**Figure 3:**
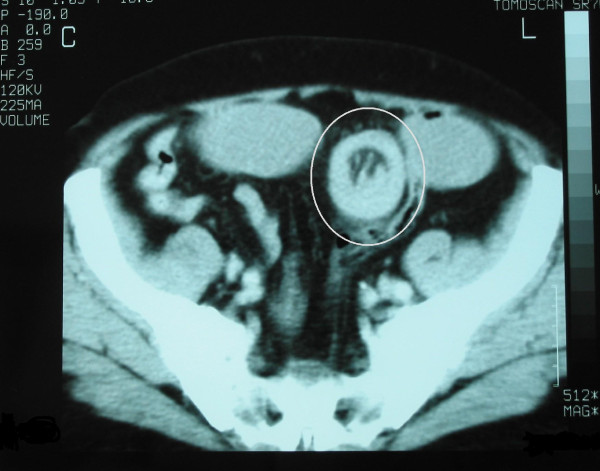
**Abdominal CT scan**. A loop of small intestine (that very probably belongs in the proximal part of ileum) in the left lesser pelvis, with abnormal wall thickening (approximately 10 mm), and a signet-ring-like unilateral thickening (marked by circle).

**Figure 4:**
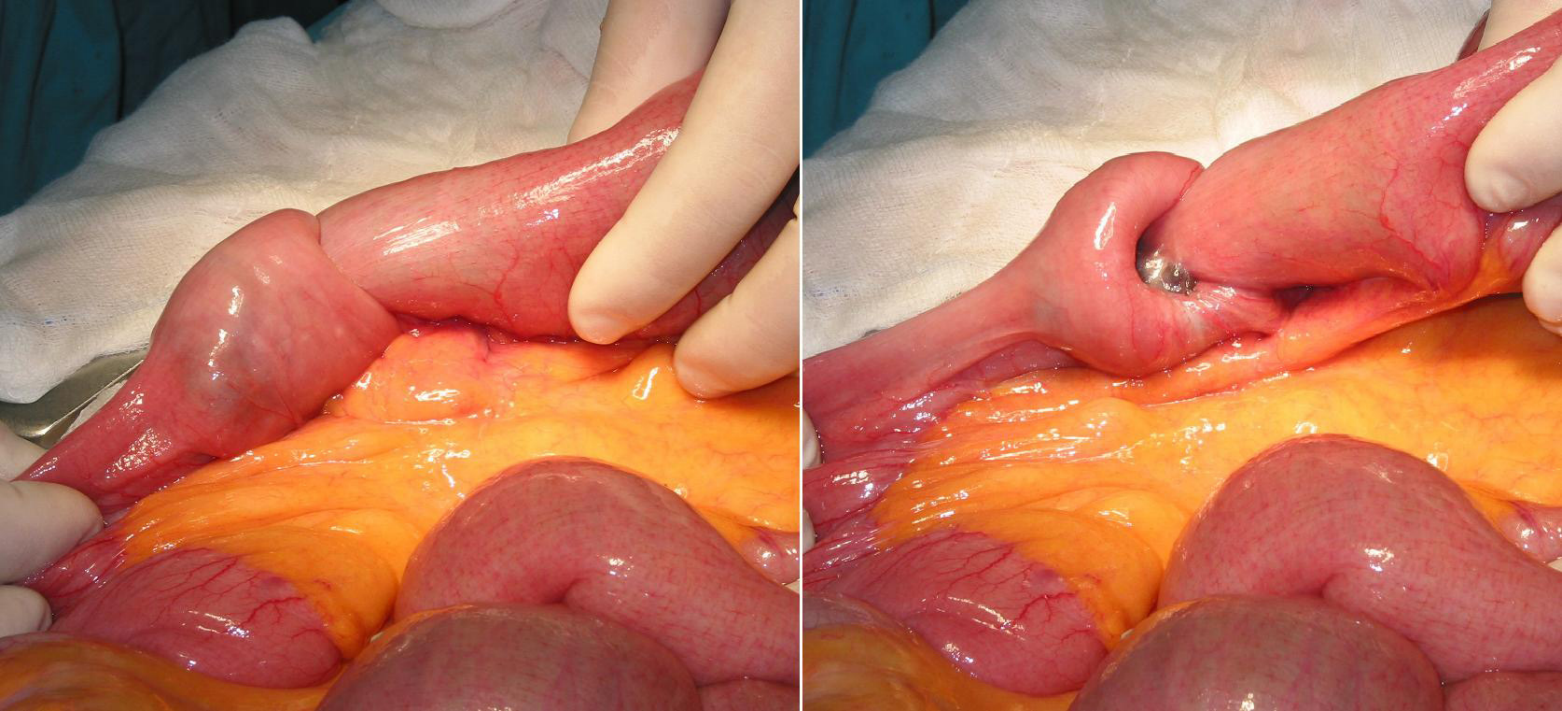
**Double jejuno-jejunal intussusception found at laparotomy**.

**Figure 5:**
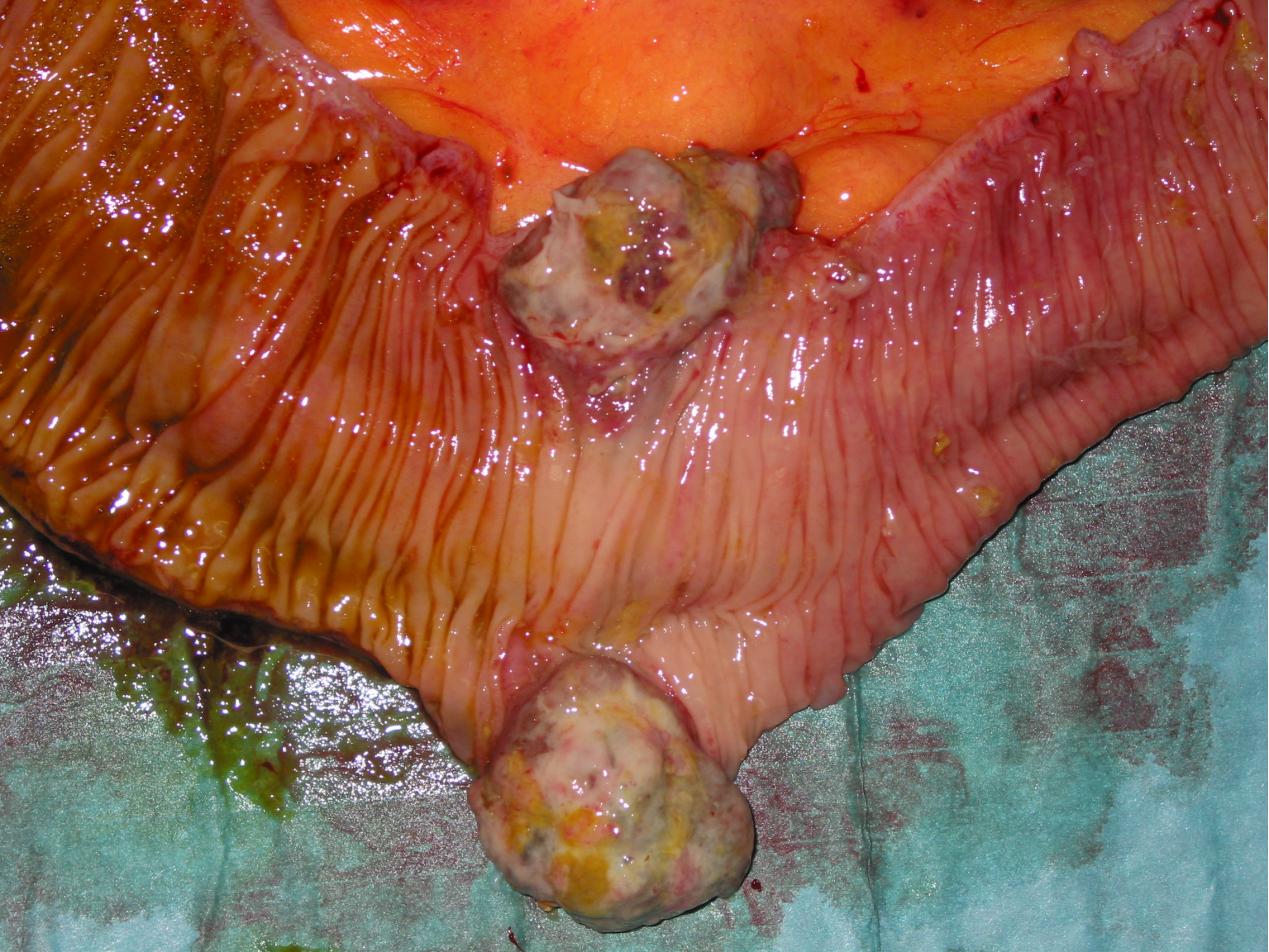
**Specimen after resection, opened, depicting the tumor**.

An 80-year-old Caucasian man was admitted to an internal medicine department at our hospital complaining of acute abdominal pain, no passage of flatus or stool, and vomiting. He was transferred to our department with the diagnosis of a probable bowel obstruction. His prior history revealed a skin lesion excised two years ago, with a histologic diagnosis of melanoma. Plain abdominal radiographs showed no air-fluid levels and an abdominal CT scan showed bowel obstruction with dilatation of his stomach and his small bowel full of liquid up to his proximal ileum. An intestinal loop with an abnormally thick wall (approximately 10 mm) was also observed. This unilateral, signet-ring-like thickening of his intestinal wall was, according to the radiologist, suggestive of enteric intussusception (Figure 3). A double (that is, the intussuscepted part was doubly imbricated) jejuno-jejunal intussusception was found at laparotomy, caused by an intraluminal mass at the terminal part of his jejunum (Figure 4). After manual reduction, the part of his jejunum with the intussusception was resected (Figure 5) and the continuity was re-established with an end-to-end anastomosis. No gross mesenteric lymphadenopathy or hepatic masses were observed. Histological examination demonstrated metastatic polypoid melanoma of the small bowel.

## Discussion

Barbette [3] was the first person to refer to intussusception in 1674. The first successful operation to a child with intussusception was carried out in 1871 by Sir Jonathan Hutchinson [4]. More than a century before this incident, Cornelius Henrik Velse operated on an adult with a similar problem which is described in "mutuo intestinorum ingressu" [2]. More details were given in 1789 by John Hunter. Hunter described three incidents, one regarding a child of nine months and two probably regarding adults, although age is not mentioned [2].

Intussusception in adults is an uncommon situation that represents 5% of the total incidents of intussusception and constitutes the cause for 1% of intestinal obstructions [5]. The usual initial clinical signs are those of bowel obstruction while the diagnosis, in contrast with children, is difficult and in almost 50% of the cases it is established intra-operatively [6]. In a simple abdominal radiograph the findings are not disease-specific, and in the radiological examination with barium (provided that the state of health of the patient allows it) the characteristic image of a corkscrew is seen. Ultrasound examination provides minimal help in adult cases, whereas it is an important diagnostic aid in children. A CT scan of the abdomen is perhaps the method with the highest diagnostic sensitivity. In transverse cuts it shows a "target" or "doughnut" sign while in the oblong cuts it shows the image of a pitchfork [6,7]. The two patients presented in our paper arrived at the hospital with bowel obstruction and in the first case the diagnosis of intussusception was established intra-operatively, while in the second case the diagnosis was based on abdominal CT findings.

Thus, in 50% of intussusception cases in adults, the causes are benign lesions such as fibromas, lipomas, adenomas and Meckel's diverticula [2,8,9]. In the remaining 50% the causes are primary tumor metastases to the gastrointestinal tract, especially melanoma which has two predominant forms in the intestine. The most common form is that of multiple sub-mucosal implants. These nodules tend to extend intraluminally as they grow, leading to gradual obstruction of the bowel lumen. Such lesions often ulcerate, resulting in occult or acute blood loss [10]. The other, less common, lesion is polypoid and often serves as the lead point for intussusception [6,9]. Regarding our second patient, the sub-mucosal implants caused intussusception when they increased in size. Metastatic breast cancer is the second most frequent malignant cause of intussusception in adults, demonstrating usually the histological type of lobular carcinoma and located in the colon and in the rectum [10-13]. In our first patient, the cause was metastatic invasive lobular carcinoma of the breast in the ileum, a condition which, to the best of our knowledge, has not been previously reported in the literature. Although there is no consensus regarding the "proper" treatment of intussusception in adult patients, there is total agreement regarding the need of laparotomy [14]. If the cause is a tumor-like lesion, resection of the affected part of the intestine and an end-to-end anastomosis are required [15-17]. This therapeutic approach was followed in our two patients during laparotomy.

## Conclusion

The pre-operative diagnosis of the cause of small bowel intussusception is difficult in adults. Although abdominal CT scanning provides the most reliable indications, it is laparotomy that establishes the diagnosis of intussusception, and the histological examination that determines the cause. A history of prior malignancy should result in the suspicion of a metastasis as a possible cause of intussusception.

## Consent

Written informed consent was obtained from both patients for publication of this case report and any accompanying images. Copies of the written consent are available for review by the Editor-in-Chief of this journal.

## Competing interests

The authors declare that they have no competing interests.

## Authors' contributions

CS was the attending surgeon on the first case. AK was the attending surgeon on the second case and author of the initial draft. NA assisted on the operation of the second case and collected the bibliographical data. SP wrote the final manuscript. AP performed the histologic examination on both cases. TG is the Head of the Department. All authors have read and approved the final manuscript.
